# Synergistic roles of NFATc1 and c-Jun in immunomodulation

**DOI:** 10.1016/j.bbrep.2025.102137

**Published:** 2025-07-07

**Authors:** Bo Cao, Renqian Wei

**Affiliations:** Department of Orthopedics Center, Affiliated Qingyuan Hospital, The Sixth Clinical Medical School, Guangzhou Medical University, Qingyuan People's Hospital, Qingyuan, China

**Keywords:** NFATc1, c-Jun, Immune regulation, Transcription factors, AP-1 complex, Synergistic interaction, Inflammatory response

## Abstract

The immune system relies on precise regulatory mechanisms, with transcription factors being central to modulating immune responses. NFATc1 and c-Jun are key transcription factors that influence both adaptive and innate immunity. This review provides a comprehensive examination of the individual and synergistic roles of NFATc1 and c-Jun in immune regulation. We delve into their molecular mechanisms, regulatory pathways, and the latest experimental approaches used to study these interactions. Furthermore, we explore the therapeutic potential of targeting these transcription factors, highlighting their clinical implications in treating immune-related diseases. This analysis underscores the critical importance of NFATc1 and c-Jun, paving the way for innovative and better therapeutic strategies and future research in immune regulation research.

## Highlights

• **NFATc1 and c-Jun as key regulators**: NFATc1 and c-Jun are pivotal transcription factors that regulate immune cell activation and differentiation, particularly in T-cells.
• **Mechanisms of cooperation**: Both transcription factors bind to overlapping DNA regions, and their interaction is essential for modulating the expression of cytokines, such as IL-2 and TNF-α.
• **Immune modulation in T-cells and beyond**: NFATc1 and c-Jun regulate immune responses not only in T-cells but also in other immune cells.
• **Pathological relevance**: Aberrant NFATc1 and c-Jun activity is implicated in autoimmune diseases, cancer, and chronic inflammation.
• **Experimentalinsights and therapeutic prospects**: In vitro and in vivo models have demonstrated the critical interactions between NFATc1 and c-Jun.

## 1 Introduction

The immune system is a complicated network of cells, tissues, and organs working in concert to defend the body against pathogens and maintain homeostasis [[1], [2], [3]]. Central to this intricate system are various regulatory mechanisms that ensure precise and appropriate immune responses [4,5]. Transcription factors play a important role in immune regulatory processes, acting as molecular switches that control the expression of genes involved in immune function [[6], [7], [8]].

Among the myriad of transcription factors, NFATc1 and c-Jun are also particularly significant [9,10]. NFATc1 (Nuclear Factor of Activated T-cells) is a member of the NFAT family, which is impotant for the development, differentiation, and function of immune cells, including T cells, B cells, and macrophages [11,12]. NFATc1 regulates gene expression by translocating to the nucleus upon activation, where it binds to specific DNA sequences and modulates transcription [13,14].

Similarly, c-Jun, a component of the Activator Protein-1 (AP-1) transcription factor complex, also plays a role in immune regulation [15]. It is involved in cellular proliferation, differentiation, and apoptosis, and is important in the response to stress signals and inflammatory stimuli [16]. c-Jun exerts effects by dimerizing with other proteins such as c-Fos, forming the AP-1 complex, which then binds to DNA and influences gene expression [17].

The synergistic interaction between NFATc1 and c-Jun is of interest, as it can impact immune responses [18]. These transcription factors often cooperate to regulate the expression of genes involved in immune activation and tolerance, thereby shaping the overall immune response [19]. Understanding the mechanisms of their interaction is vital for elucidating the complexities of immune regulation [20,21]. Understanding the mechanisms of their interaction is essential for elucidating immune regulation complexities and identifying potential therapeutic targets for immune-related disorders.

Transcriptional activity of NFATc1 and c-Jun is not solely determined by their DNA-binding capacities but is also modulated by coactivators [9]. Coactivators serve as important mediators that enhance transcription factor function, bridging interactions between transcription factors and the basal transcriptional machinery [22,23]. Key coactivators such as CBP/p300, SRC family members, and MED proteins have been implicated in NFATc1 and c-Jun-mediated gene regulation [9,24]. CBP/p300, for instance, provides histone acetyltransferase activity, facilitating chromatin remodeling and promoting transcriptional activation [25]. Additionally, coactivators modulate the recruitment of NFATc1 and c-Jun to specific gene loci, thereby influencing immune cell fate and response dynamics [26]. Understanding how coactivators integrate with NFATc1 and c-Jun is essential for developing targeted therapeutic strategies that modulate immune function.

This review aims to provide a overview of the roles of NFATc1 and c-Jun in immunomodulation, detailing their individual and combined functions. We will explore the molecular mechanisms underlying their synergistic interaction, highlight experimental approaches used to study these transcription factors, and discuss the therapeutic potential of targeting NFATc1 and c-Jun in immune-related diseases. By synthesizing current knowledge and identifying gaps in understanding, this review seeks to pave the way for future research and innovative therapeutic strategies in immune regulation.

## 2 NFATc1 in immune regulation

**Fig. 1:**
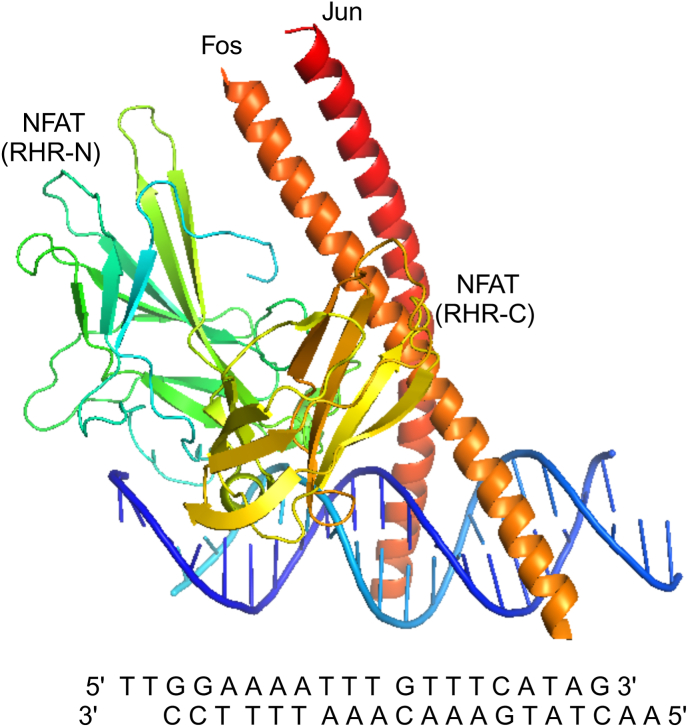
The MOLSCRIPT representation of the NFAT–AP-1–DNA complex [122]. The N-terminal domain (RHR-N) of the NFAT RHR is green; the C-terminal domain (RHR-C) is yellow; Fos is orange; Jun is red. Strands and helices are labelled.

**Fig. 2:**
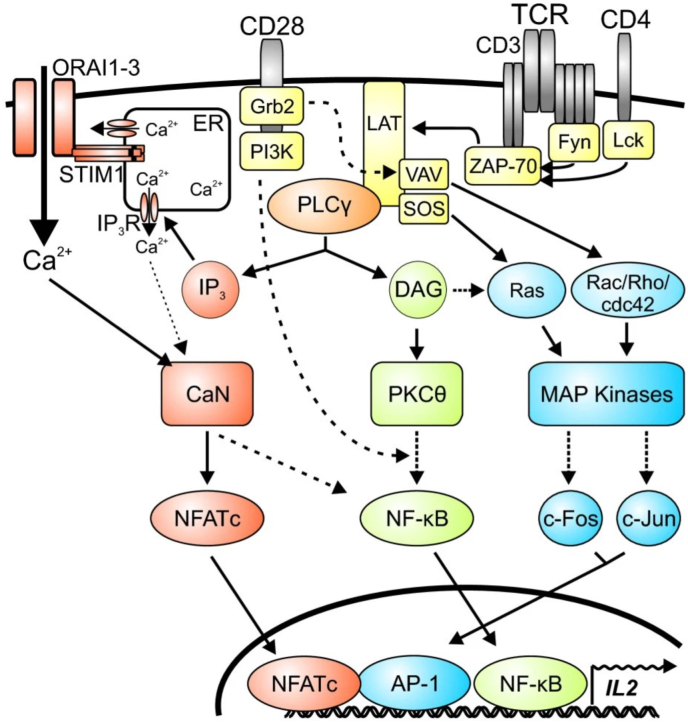
Schematic overview about TCR-dependent signalling pathways [36]. Engagement of TCRs and costimulatory CD28 receptors promote signalling cascades of kinases and adaptor proteins (yellow). They trigger pathways resulting in the activation of the transcription factors NFATc (red), NF-κB (green) and AP-1 (blue).

NFATc1, a member of the Nuclear Factor of Activated T-cells (NFAT) family, is a transcription factor characterized by its Rel homology domain (RHD) responsible for DNA binding [[27], [28], [29], [30], [31]]. Additionally, it also features regulatory domains that control its activation and subcellular localization [[32], [33], [34]]. NFATc1's structure(Fig. 1) allows it to form complexes with other transcription factors, thereby influencing gene expression synergistically [32,34]. NFATc1 is activated by calcium signaling pathways [35]. Upon T-cell receptor (TCR) engagement(Fig. 2), intracellular calcium levels rise, leading to the activation of the phosphatase calcineurin [28,36]. Calcineurin dephosphorylates NFATc1, causing its conformational change and subsequent translocation from the cytoplasm to the nucleus [11,32]. Once in the nucleus, NFATc1 binds to DNA at specific response elements, regulating the transcription of target genes [37].

The NFATc1 gene is regulated at the chromosomal level within a complex locus enriched with regulatory elements. It is located on human chromosome 18q23 and consists of multiple alternative promoters and enhancers that contribute to its dynamic expression in various immune cell types [38,39]. The transcriptional regulation of NFATc1 is further modulated by its remote enhancer, which resides several kilobases upstream of the gene and plays a crucial role in immune cell-specific expression [40,41]. This remote enhancer interacts with the NFATc1 promoter through chromatin looping, facilitating enhancer-promoter communication that enhances gene transcription [42]. The enhancer region is enriched with binding sites for key transcription factors, including NF-κB, AP-1, and additional NFAT family members, thereby integrating signals from multiple pathways to fine-tune NFATc1 expression [12]. Epigenetic modifications, such as histone acetylation and DNA demethylation, further regulate the activity of this enhancer, ensuring cell-type-specific expression patterns [43,44]. In activated T cells, the enhancer undergoes rapid chromatin remodeling to enable robust NFATc1 transcription, whereas in resting cells, repressive histone marks limit its activity [45].

NFATc1 is importantfor T-cell activation and function [46,47]. Upon TCR engagement, the activation of NFATc1 is impaortant for the transcription of genes involved in T-cell proliferation, differentiation, and effector functions [48]. The interaction of NFATc1 with other transcription factors like AP-1 (of which c-Jun is a component) enhances its ability to modulate gene expression [49]. In T-cells, NFATc1 regulates several critical genes, including those encoding cytokines (such as IL-2), cell surface receptors (such as CD25), and other proteins crucial for T-cell function and survival [46,50]. These genes are vital for the immune response, influencing T-cell proliferation, differentiation, and effector functions [51].

Beyond T-cells, NFATc1 is active in other immune cells, including macrophages, dendritic cells, and B cells (Table 1). In macrophages, NFATc1 influences the production of pro-inflammatory cytokines and the expression of co-stimulatory molecules, thus modulating the immune response [[52], [53], [54]]. In dendritic cells, NFATc1 plays a role in antigen presentation and the activation of T-cells [55]. These functions highlight NFATc1's broad regulatory capacity across different cell types within the immune system.

**Table 1:** Different functions of NFAT 1 transcription factors in innate cells [157].

Cell type	Function
DCs	Regulation of the DC life cycle/apoptosis of terminally differentiated DCs
Macrophages	Regulation of IL-6, IL-10, and IL-12 and TNF-α expression
Dendritic cells	Activation of T-cells
Eosinophils	Degranulation, cytokine release, apoptosis
NK cells	Role in cytotoxicity and cell proliferation

NFATc1 also serves a key regulator of cytokine production, influencing both pro-inflammatory and anti-inflammatory responses [56,57]. By controlling the expression of cytokines such as IL-2, IL-4, IL-10, and TNF-α, NFATc1 modulates the magnitude and nature of the immune response [23,[58], [59], [60], [61]]. This regulatory capability makes NFATc1 a important player in maintaining immune homeostasis and responding to infections [11,62,63].

NFATc1's function is closely related to that of NFATc2, another NFAT family member [64]. While both factors contribute to immune regulation, NFATc1 is primarily often associated with immediate-early responses in T-cell activation, whereas NFATc2 plays a role in sustaining these responses and modulating long-term immune activity [65,66]. Although NFATc1 and NFATc2 can compensate for each other in certain immune contexts, but their distinct temporal and functional characteristics enable fine-tuned immune regulation [67]. The interplay between NFATc1 and NFATc2 ensures a balanced immune response, preventing excessive activation that could lead to autoimmunity [23].

NFATc1's dysregulation has been implicated in several autoimmune diseases, where its aberrant activation leads to the inappropriate activation of immune cells and the production of autoantibodies [11,68,69]. Conditions such as rheumatoid arthritis, systemic lupus erythematosus, and multiple sclerosis have been linked to altered NFATc1 activity [11,70]. In cancer, NFATc1 can influence tumor growth and progression by regulating the expression of genes involved in cell proliferation and survival [[71], [72], [73]]. Additionally, NFATc1's role in chronic inflammatory conditions, such as inflammatory bowel disease and chronic obstructive pulmonary disease, underscores its importance in both immune regulation and pathology [[74], [75], [76]]. Targeting NFATc1 in these diseases offers potential therapeutic avenues, highlighting the need for further research into its regulatory mechanisms and interactions. This comprehensive examination of NFATc1's roles across various immune cells and conditions underscores its critical importance in immune regulation and its potential as a therapeutic target.

## 3 c-Jun in immune regulation

**Fig. 3:**
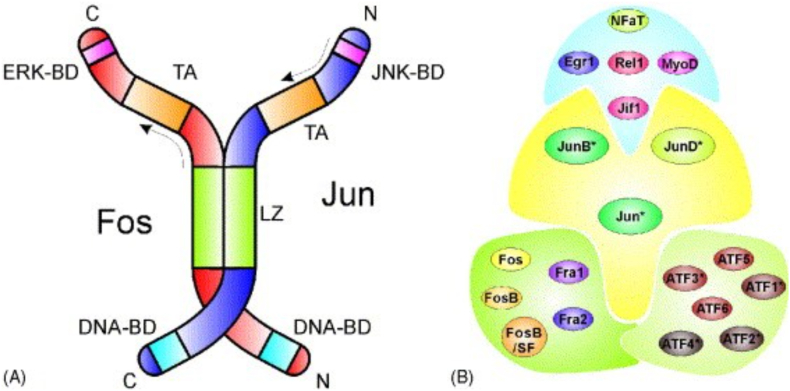
AP1 transcription factor dimers [61]. (A) The classical c-Jun/c-Fos heterodimer, containing the DNA binding domains (DNA-BD), the leucine zipper (LZ) region, the transactivating module (TA), and the binding domains for the mitogen-activated protein kinases, ERK (ERK-BD) in the case of c-Fos, and JNK (JNK-BD) for c-Jun. (B) A summary of dimer-forming interactions between different groups of AP1 transcription factor families. Members of the c-Jun family can form various heterodimers with the adjacent c-Fos family (left), the ATF family (right) or a large group of transcription factors including Egr1, Rel1, Zif1, MyoD or NFAT. All Jun family members and most ATF family members, marked with ∗.

c-Jun is a important component of the Activator Protein-1 (AP-1) transcription factor complex(Fig. 3) and belongs to the Jun protein family [77,78]. It contains a basic leucine zipper (bZIP) domain, which facilitates DNA binding and dimerization with other AP-1 proteins [57,77,79]. The structure of c-Jun is important for its ability to regulate gene expression and respond to cellular signals. c-Jun activation is primarily mediated by the c-Jun N-terminal kinase (JNK) signaling pathway [[80], [81], [82]]. Upon activation by stress signals, cytokines, or growth factors, JNK phosphorylates c-Jun at specific serine residues, enhancing its transcriptional activity [80,81,83]. This phosphorylation leads to increased stability and DNA binding affinity of c-Jun, enabling it to regulate target gene expression effectively [84,85].

c-Jun functions within the AP-1 complex by dimerizing with other members of the Jun family or the Fos family [69,86,87]. These dimers bind to specific DNA sequences known as TPA-response elements (TRE) within the promoter regions of target genes, facilitating transcriptional regulation [9,[88], [89], [90]]. The AP-1 complex binds to DNA through its bZIP domain, influencing the transcription of genes involved in proliferation, differentiation, and apoptosis [[91], [92], [93]]. The composition of the AP-1 dimer determines its binding affinity and transcriptional activity, thereby finely tuning gene expression in response to various stimuli [94,95]. While other AP-1 factors, such as BATFs, also contribute to immune regulation. c-Jun plays a distinct and central role in modulating gene expression across multiple immune processes [23,96].

c-Jun plays a important role in T-cell activation and differentiation [15,97]. Upon TCR engagement, c-Jun is rapidly phosphorylated and activated, promoting the transcription of genes necessary for T-cell proliferation and effector function [9]. This includes cytokines, growth factors, and other molecules essential for T-cell responses [57,98,99]. c-Jun interacts with NFATc1 to regulate gene expression synergistically [100,101]. This interaction enhances the transcription of target genes involved in T-cell activation, providing a robust and coordinated response [102,103]. The synergy between c-Jun and NFATc1 is vital for optimal T-cell function and effective immune responses [104].

Beyond T-cell regulation, c-Jun is a important regulator of inflammatory responses [105]. It controls the expression of various pro-inflammatory genes, including TNF-α and matrix metalloproteinases (MMPs), which are involved in tissue remodeling and inflammation [106]. By modulating these genes, c-Jun influences the intensity and duration of inflammatory responses [107,108]. Aberrant c-Jun activity is implicated in several inflammatory diseases [83]. Its dysregulation can lead to chronic inflammation and tissue damage, contributing to conditions such as rheumatoid arthritis, psoriasis, and inflammatory bowel disease [[109], [110], [111], [112]]. Understanding c-Jun's role in these diseases may offer new therapeutic targets. c-Jun is also involved in cancer and other chronic diseases [83,113,114]. It can promote oncogenesis by regulating genes involved in cell proliferation, survival, and metastasis [115]. Additionally, c-Jun's role in chronic inflammatory conditions links it to the progression of diseases such as atherosclerosis and fibrosis [116,117]. Targeting c-Jun in these contexts holds potential for novel therapeutic interventions. This detailed examination of c-Jun's roles underscores its critical importance in immune regulation and pathology, highlighting its potential as a therapeutic target in various diseases.

## 4 Synergistic interaction between NFATc1 and c-Jun

The synergy between NFATc1 and c-Jun is grounded in their ability to physically interact and form a functional complex [118]. Structural studies have revealed that NFATc1 possesses a Rel homology domain (RHD) that facilitates its binding to DNA, while c-Jun contains a basic leucine zipper (bZIP) domain essential for dimerization and DNA binding [119,120]. When these proteins co-localize on DNA, their structural compatibility enhances their combined binding affinity and stability on the target DNA sequences [121,122]. Cooperative DNA binding by NFATc1 and c-Jun occurs through direct protein-protein interactions and the formation of a stable transcriptional complex at specific promoter regions [123]. This cooperative binding is facilitated by the recruitment of co-activators and the remodeling of chromatin, which opens up the DNA and allows both transcription factors to access their binding sites more efficiently [124]. The presence of NFATc1 can induce conformational changes in the DNA that enhance the binding of c-Jun, and vice versa, leading to a more robust transcriptional activation [32,125].

Coactivators play a pivotal role in stabilizing the NFATc1-c-Jun complex and facilitating chromatin remodeling for effective gene activation [9]. Key coactivators involved in this process include CBP/p300, PCAF, and other transcriptional mediators.CBP (CREB-binding protein) and p300 function as transcriptional coactivators with intrinsic histone acetyltransferase (HAT) activity, which is important for chromatin remodeling and gene activation [25]. These coactivators act as scaffolds that bridge NFATc1 and c-Jun, stabilizing their interaction at target promoters [126]. CBP/p300-mediated acetylation of histones loosens chromatin structure, making promoter regions more accessible to transcription factors [127]. Additionally, CBP/p300 directly acetylates NFATc1 and c-Jun, enhancing their DNA-binding affinity and transcriptional activity [128]. The recruitment of CBP/p300 to NFATc1-c-Jun target genes amplifies immune responses, particularly in T-cells, where this interaction is crucial for the expression of cytokines such as IL-2 and IL-4 [129].

PCAF (p300/CBP-associated factor) is another key coactivator that modulates NFATc1 and c-Jun activity through its HAT function and coactivator recruitment capabilities [130]. PCAF interacts with NFATc1 and c-Jun, promoting their acetylation and enhancing transcriptional synergy at immune gene promoters [131]. In T-cells, PCAF contributes to IL-2 and IFN-γ production by facilitating the formation of a transcriptionally competent NFATc1-c-Jun complex [132]. Beyond acetylation, PCAF functions as a co-scaffold, coordinating with CBP/p300 to recruit additional chromatin-modifying enzymes that enhance gene activation [133].

Other coactivators, such as SRC-1 (steroid receptor coactivator-1) and mediator complex subunits (MED proteins), further modulate NFATc1 and c-Jun transcriptional synergy [134].

Enhancing the recruitment of RNA polymerase II to NFATc1-c-Jun target genes [135]. Promoting chromatin accessibility through histone modifications and nucleosome remodeling [136]. Stabilizing transcriptional complexes at enhancer regions of immune-responsive genes [137]. In immune cells such as macrophages and dendritic cells, these coactivators regulate genes involved in inflammation and antigen presentation, broadening the impact of NFATc1-c-Jun interactions beyond T-cell biology [138,139].

In T-cells, NFATc1 and c-Jun jointly regulate a variety of genes essential for T-cell activation, differentiation, and function [140]. This includes cytokine genes such as IL-2, which are critical for T-cell proliferation and survival [26]. The coordinated regulation by NFATc1 and c-Jun ensures a synchronized transcriptional response, which is crucial for the effective activation and expansion of T-cells during an immune response [141]. The synergistic action of NFATc1 and c-Jun enhances the magnitude and specificity of T-cell responses [9]. By cooperatively binding to and activating key gene promoters, these transcription factors amplify signals that drive T-cell proliferation, differentiation into effector cells, and cytokine production [26]. This synergy not only boosts the immediate immune response but also helps in the formation of memory T-cells, which are essential for long-term immunity.

The interaction between NFATc1 and c-Jun extends beyond T-cell biology, influencing a wide range of immune cells and responses [142,143]. In macrophages, dendritic cells, and other immune cells, the NFATc1 and c-Jun complex regulates genes involved in inflammation, antigen presentation, and cell survival [49,144,145]. This broad regulatory role highlights the importance of their synergy in maintaining immune homeostasis and responding to various immune challenges. Given their critical role in immune regulation, targeting the NFATc1 and c-Jun interaction presents a promising therapeutic strategy for various diseases. Modulating this interaction could help in treating autoimmune diseases by dampening aberrant T-cell activation and reducing inflammation. In cancer, disrupting the NFATc1 and c-Jun synergy could impair tumor cell survival and proliferation [49,146]. Additionally, therapeutic interventions could aim to enhance this synergy to boost immune responses against infections and in immunotherapy settings. Understanding the molecular and functional interplay between NFATc1 and c-Jun thus opens new avenues for developing targeted therapies that can finely tune the immune response for therapeutic benefit.

## 5 Experimental approaches to study NFATc1 and c-Jun

A comprehensive understanding of NFATc1 and c-Jun interactions requires a multifaceted experimental approach that integrates cellular, molecular, and in vivo models. These methods provide important insights into the functional roles and regulatory mechanisms of these transcription factors in immune responses.

In vitro studies using cell culture models are fundamental for investigating the functions and interactions of NFATc1 and c-Jun [102]. Various immune cell lines, such as Jurkat T cells and primary T cells, are commonly used to analyze the activation and functional roles of these transcription factors [141,147,148]. Techniques such as flow cytometry, ELISA, and western blotting are employed to measure cytokine production, protein expression, and activation status of NFATc1 and c-Jun in response to specific stimuli [[149], [150], [151], [152]]. Collectively, these methods provide mechanistic insights into the signaling cascades regulating these transcription factors.

To examine the transcriptional activities of NFATc1 and c-Jun, reporter gene assays are pivotal in studying the transcriptional activity of NFATc1 and c-Jun [153]. Constructs containing NFAT or AP-1 responsive elements linked to a luciferase or GFP reporter are transfected into cells, and the reporter activity is measured to assess the transcriptional activation [154]. Coupled with mutagenesis studies, reporter assays can elucidate specific DNA-binding motifs and protein-protein interactions contributing to transcriptional regulation. Chromatin immunoprecipitation (ChIP) assays are used to determine the binding of NFATc1 and c-Jun to specific genomic regions [155]. This technique involves crosslinking proteins to DNA, shearing the chromatin, and using antibodies specific to NFATc1 or c-Jun to precipitate the protein-DNA complexes. Subsequent sequencing (ChIP-seq) or PCR analysis identifies the binding sites of these transcription factors on the genome [[156], [157], [158]]. This approach enables the identification of *cis*-regulatory elements and cooperative binding sites, offering insights into the transcriptional networks orchestrated by NFATc1 and c-Jun.

Genetically modified mouse models are indispensable for understanding the roles of NFATc1 and c-Jun in immune regulation [142,159]. These models help in studying the physiological and pathological contexts of these transcription factors in a whole organism [160]. Knockout models, where NFATc1 or c-Jun genes are selectively deleted, and knock-in models, where mutations or tags are introduced into these genes, are instrumental in studying their functions [161]. These models allow researchers to observe the effects of the absence or altered function of NFATc1 and c-Jun on immune responses, development, and disease progression [32]. Conditional knockout models, where gene deletion is restricted to specific cell types or induced at particular developmental stages, provide insights into the cell-specific roles of these transcription factors [162].

Surface Plasmon Resonance (SPR) is a powerful technique used to study the interactions between NFATc1, c-Jun, and other proteins or DNA molecules. SPR measures the binding kinetics and affinities by detecting changes in the refractive index near the sensor surface to which one interaction partner is immobilized [163]. This real-time, label-free method provides quantitative data on the strength and dynamics of molecular interactions, understanding the cooperative mechanisms between NFATc1 and c-Jun.

High-throughput sequencing techniques, such as RNA-seq and ChIP-seq [164], offer comprehensive insights into the genomic binding sites and gene expression profiles regulated by NFATc1 and c-Jun. These techniques help identify direct target genes and elucidate the broader regulatory networks controlled by these transcription factors. Proteomics approaches [165], including mass spectrometry, are employed to analyze the protein-protein interactions, post-translational modifications, and the composition of protein complexes involving NFATc1 and c-Jun.

These advanced analytical methods provide a holistic view of the molecular mechanisms underlying the functions of these transcription factors in immune regulation. Researchers, integrating these experimental approaches, can gain a deeper understanding of the synergistic roles of NFATc1 and c-Jun in immune regulation, paving the way for potential therapeutic interventions targeting these transcription factors.

## 6 Possible clinical implications

Autoimmune diseases arise from dysregulated immune responses against self-antigens, leading to chronic inflammation and tissue damage [166]. NFATc1 and c-Jun have emerged as important regulators in immune cell activation and differentiation, making them attractive targets for therapeutic intervention [26].

Current therapeutic strategies in autoimmune diseases often involve broad immunosuppressive agents, which can lead to non-specific suppression of immune function and potential side effects [167]. Targeting specific pathways such as NFATc1 and c-Jun offers the potential for more precise modulation of immune responses [168]. Cyclosporin A (CsA) and FK506 targeting NFATc1 activation have been developed and tested in preclinical models [169]. These inhibitors work by disrupting the interaction between NFATc1 and its binding partners or by inhibiting NFATc1 nuclear translocation. Inhibitors of c-Jun, such as kinase inhibitors tofacitinib targeting upstream kinases involved in c-Jun activation, are under investigation [170]. These inhibitors aim to block the phosphorylation and subsequent activation of c-Jun, thereby modulating its transcriptional activity. Given the synergistic roles of NFATc1 and c-Jun in immune regulation, combination therapies targeting both transcription factors simultaneously may provide enhanced efficacy compared to single-agent therapies. Combination approaches could potentially achieve greater control over autoimmune responses while minimizing off-target effects [171,172]. Ongoing research focuses on improving the specificity and safety profiles of NFATc1 and c-Jun inhibitors. Additionally, efforts are directed towards identifying biomarkers that can predict patient response to these targeted therapies, thereby enabling personalized treatment approaches in autoimmune diseases.

NFATc1 and c-Jun also play important roles in cancer progression, particularly within the tumor microenvironment where they contribute to immune evasion, metastasis, and resistance to therapy [173,174]. In various cancers, NFATc1 promotes tumor growth and survival by regulating genes involved in proliferation, angiogenesis, and immune suppression [64,72,175]. Inhibiting NFATc1 activity holds promise as a therapeutic strategy to disrupt these processes [176]. c-Jun is implicated in oncogenic transformation, tumor invasion, and resistance to chemotherapy [177,178]. Targeting c-Jun signaling pathways, either through direct inhibition or upstream kinase inhibition, represents a potential avenue for cancer therapy. Strategies combining NFATc1 and c-Jun inhibitors with immunotherapy are being explored to enhance anti-tumor immune responses and improve treatment outcomes in cancer patients. Clinical trials investigating NFATc1 and c-Jun inhibitors alone or in combination with standard therapies are underway. These trials aim to validate the therapeutic potential of targeting these transcription factors in specific cancer types and define optimal treatment regimens.

Chronic inflammatory diseases, such as rheumatoid arthritis and inflammatory bowel disease, are characterized by persistent inflammation and tissue damage mediated by dysregulated immune responses [179,180]. NFATc1 and c-Jun are key regulators in the inflammatory pathways, offering potential targets for therapeutic intervention [35,181]. Selective inhibitors of NFATc1 are being developed to suppress its activity in immune cells involved in chronic inflammation [169]. These inhibitors aim to mitigate inflammatory responses while preserving essential immune functions [169]. In certain contexts, activating c-Jun signaling pathways may promote resolution of inflammation or tissue repair [170,182]. Strategies to selectively activate c-Jun in a controlled manner are under investigation for their therapeutic potential in inflammatory diseases. The development of biomarkers to stratify patients based on NFATc1 and c-Jun activity levels may facilitate personalized treatment approaches. This precision medicine approach aims to optimize therapeutic efficacy while minimizing adverse effects. Novel therapeutic modalities, including gene editing technologies and RNA-based therapies targeting NFATc1 and c-Jun, are being explored for their potential in modulating immune responses in inflammatory diseases.

NFATc1 and c-Jun represent promising targets for therapeutic intervention across a spectrum of immune-related diseases, from autoimmune disorders to cancer and chronic inflammation [183,184]. Advances in our understanding of the molecular mechanisms underlying their roles in disease pathogenesis continue to drive the development of innovative therapies aimed at improving patient outcomes. Future research efforts should focus on refining these therapeutic strategies and translating preclinical findings into clinical applications, thereby realizing the therapeutic potential of targeting NFATc1 and c-Jun in immune regulation.

## 7 Findings and future directions

NFATc1 and c-Jun are important transcription factors that intricately modulate immune responses through their synergistic roles in gene regulation and cellular signaling pathways [32]. This review consolidates significant findings that underscore their importance in immune regulation. NFATc1 and c-Jun collaboratively orchestrate T cell activation and differentiation by regulating the expression of key cytokines and surface receptors involved in immune responses [49]. NFATc1 and c-Jun influence immune tolerance mechanisms and their dysregulation contributes to the pathogenesis of autoimmune diseases [185]. Understanding their precise roles in maintaining immune homeostasis is crucial for developing targeted therapies. Both transcription factors play critical roles in inflammatory signaling pathways across various immune cell types, influencing the production of inflammatory mediators and the resolution of inflammation. NFATc1 and c-Jun contribute to the tumor microenvironment by regulating immune evasion mechanisms, making them potential targets for cancer immunotherapy. Targeting NFATc1 and c-Jun pathways holds promise for treating autoimmune diseases, inflammatory disorders, and certain cancers. However, challenges remain in achieving specificity and minimizing off-target effects.

Despite significant progress, several knowledge gaps warrant further investigation: Elucidating context-specific functions of NFATc1 and c-Jun in different immune cell subsets and disease states is essential. This includes understanding their roles in tissue-specific immunity and how their activities are regulated spatially and temporally. Investigating the mechanisms underlying the crosstalk between NFATc1 and c-Jun pathways, particularly in the context of immune cell activation and differentiation, remains a key area of interest. This includes identifying shared regulatory elements and feedback loops that fine-tune their activities. Further validation of findings from in vitro studies using robust in vivo models is necessary to better mimic the complexity of immune responses and disease pathogenesis. This includes the development of genetically engineered mouse models and patient-derived xenograft models. Identifying reliable biomarkers associated with NFATc1 and c-Jun activities could aid in patient stratification for targeted therapies. Developing predictive tools to assess treatment response and potential resistance mechanisms is critical for optimizing clinical outcomes.

To address these knowledge gaps, future research efforts should focus on the following directions: Utilize single-cell transcriptomics and epigenomics approaches to dissect cell-specific functions of NFATc1 and c-Jun in immune cells under normal and pathological conditions. Conduct mechanistic studies to unravel the molecular mechanisms underlying NFATc1 and c-Jun interactions with other transcription factors, chromatin modifiers, and signaling pathways. Validate the therapeutic potential of targeting NFATc1 and c-Jun in preclinical models of autoimmune diseases, chronic inflammation, and cancer. Explore combination therapies and assess long-term safety and efficacy. Translate preclinical findings into clinical trials to evaluate the efficacy of NFATc1 and c-Jun inhibitors in patients. Incorporate biomarker-driven approaches to personalize treatment strategies and monitor therapeutic responses. Leverage emerging technologies such as CRISPR/Cas9 gene editing, high-resolution imaging, and computational modeling to advance our understanding of NFATc1 and c-Jun biology and their roles in disease pathogenesis.

Further exploration of NFATc1 and c-Jun in immune regulation promises to uncover new insights into disease mechanisms and therapeutic strategies. Addressing these research priorities will not only deepen our understanding of immune biology but also pave the way for novel treatments that could benefit patients with immune-related disorders and cancer.

## 8 Conclusion

This review has comprehensively explored the synergistic roles of NFATc1 and c-Jun in immune regulation, shedding light on their intricate mechanisms, experimental insights, and therapeutic prospects. Advancements in technology, including high-throughput sequencing, single-cell analysis, and advanced imaging techniques, will be instrumental in unraveling the complexities of NFATc1 and c-Jun signaling networks. By harnessing the full potential of NFATc1 and c-Jun as therapeutic targets, we can envision a future where targeted immunotherapies transform the landscape of immune-related disorders and cancer treatment.

## CRediT authorship contribution statement

**Bo Cao:** Writing – original draft, Writing – review & editing. **Renqian Wei:** Writing – review & editing, Supervision.

## Consent to publication

All authors consent to the publication of this manuscript, titled Synergistic Roles of NFATc1 and c-Jun in Immunomodulation, and affirm that the work described was original research that has not been published previously, and not under consideration for publication elsewhere, in whole or in part.

## Statement of ethics

Ethics approval for this study was not apply in this study. Clinical trial number: not applicable.

## Data availability statement

The authors confirm that the data supporting the findings of this study are available within the article. Further enquiries can be directed to the corresponding author.

## Funding sources

This work was supported China Scholarship Committee 202008440406.

## Declaration of competing interest

The authors Bo Cao, PhD, and Renqian Wei, PhD, declare that they have no conflicts of interest related to the publication of this review paper titled Synergistic Roles of NFATc1 and c-Jun in Immunomodulation. The research presented in this manuscript is independent of any financial or personal relationships that could influence the results or interpretations. No external funding was received for this work, and there are no relevant affiliations or financial interests to disclose.
